# Attitudes of Psychiatric Nurses about the Request for Euthanasia on the Basis of Unbearable Mental Suffering(UMS)

**DOI:** 10.1371/journal.pone.0144749

**Published:** 2015-12-23

**Authors:** Marc De Hert, Liesbet Van Bos, Kim Sweers, Martien Wampers, Jan De Lepeleire, Christophe U. Correll

**Affiliations:** 1 University Psychiatric Centre Z.org KU Leuven, KU Leuven Department of Neurosciences, Leuven, Belgium; 2 Academic Center for General Practice, Leuven, Belgium; 3 Department of Public Health and Primary Care, KU Leuven, Kortenberg, Belgium; 4 The Zucker Hillside Hospital, Glen Oaks, New York, United States of America; Medical University of Vienna, AUSTRIA

## Abstract

**Introduction:**

When psychiatric patients express a wish for euthanasia, this should first and foremost be interpreted as a cry for help. Due to their close day-to-day relationship, psychiatric nurses may play an important and central role in responding to such requests. However, little is known about nurses’ attitudes towards euthanasia motivated by unbearable mental suffering.

**Objectives:**

The aim of this study was to provide insight into the attitudes and actions taken by psychiatric nurses when confronted with a patient’s euthanasia request based on unbearable mental suffering (UMS).

**Method:**

A questionnaire was sent to 11 psychiatric hospitals in the Flemish part of Belgium.

**Results:**

The overall response rate was 70% (N = 627). Psychiatric nurses were frequently confronted with a request for euthanasia, either directly (N = 329, 53%) or through a colleague (N = 427, 69%). A majority (N = 536, 84%) did not object to euthanasia in a psychiatrically ill population with UMS. Confounding factors were the psychiatric diagnosis and the type of ward where the nurses were working. Most participants acknowledged a lack of knowledge and skills to adequately address the euthanasia request (N = 434, 71%). Nearly unanimously (N = 618, 99%), study participants indicated that dealing with euthanasia requests and other end-of-life issues should be part of the formal training of nurses.

**Conclusion:**

The results highlight the need for ethically sound and comprehensive provision of care. Psychiatric nurses play an important role in dealing with the complex issue of requests for euthanasia. There is also a need for education, training and clear guidelines on the level of health care organizations.

## Introduction

The Belgian legislation on euthanasia, passed on May 28^th^ 2002,defines euthanasia as an intentional life-ending act by a physician at a persons’ explicit request under specific conditions [1]. On February 13^th^ 2014 an amendment of the law has made euthanasia a possibility for minors [1]. The patient requesting euthanasia has to be a competent adult who has unbearably suffering from a serious medical condition, including psychiatric illnesses, with no therapeutic perspective nor prospect of alleviating suffering. According to the Belgian law, unbearable suffering can be physical and/or mental. The patients’ request has to be voluntary and well-considered, implying adequate decision making capacity. In a terminal stage of illness one independent physician is required to examine the patient and give advice regarding the request to the physician who received the initial request. If the patient is not in a terminal stage two additional requirements need to be met. At least one month must pass between the written euthanasia request and life determination, and a second independent physician is required to examine the patient and give once again advice regarding the request. This physician needs to be a psychiatrist in case of psychiatric illnesses. When euthanasia is requested by a minor, the procedure is equal except for a required consent of parents or legal guardians [1].

According to annual reports of the federal control and evaluation commission on euthanasia in Belgium, requests for euthanasia based on UMS are rapidly increasing. In a period of seven years (2002–2009) 1,5% (52) deaths were due exclusively to neuropsychiatric disorders. In the annual report from 2010 till 2011 the requests based on UMS were already 58 (2.8%). A retrospective study showed that over a time period from 2007 till 2010 hundred requests were based on UMS, where from 48 were accepted and 35 died by euthanasia [2]. In the general population the number of advanced directives regarding euthanasia have increased by 60% (20,000) last year.

A request for euthanasia based on UMS is a topic that has raised important clinical and ethical considerations [3; 4]. Dealing with euthanasia requests based on UMS should be a on cautious base within the existing legal framework. One needs to be absolutely certain that this intervention is the last resource for the patient [5]. The process towards euthanasia can lead to increased demoralization [1]. While a request for euthanasia by a psychiatrically ill patient can also be a cry for help [6; 7]. Paradoxically a request for euthanasia in a psychiatric patient is not always related to hopelessness. When the request is adequately addressed and the patient is given the ability to talk extensively, this may lead to recognition and a possible decrease of suffering. It is not uncommon that while the request is taken serious and given enough attention that up to 50% of patients put their request ‘on hold’ [4; 7; 8]. A demand for euthanasia is there for not solely a request for action, but also provides meaningful material that a mental health care worker can utilize as part of therapeutic process. By integrating the request as part of holistic care, patients perspectives of living with the disease and its consequences may contribute to integrated acceptance [2; 9].

Recognizing, that life is not being considered to have value and a purpose for the patient requesting euthanasia, is out of the professional comfort zone for every mental health worker [10]. Also for psychiatric nurses, who are trained to deal with suicidal ideation with a focus on life and hope. Even so, with the knowledge that recognition of a euthanasia request can lead to a meaningful therapeutic process with acceptance, should motivate every mental health worker in dealing with these kind of requests. Different aspects of recognition such as listening and presence are core tasks of psychiatric nurses. In inpatient units, nurses are the key care providers for a patient. There for could have a unique position in dealing with end of life questions, such as requests for euthanasia [10; 11]. Despite this unique position of psychiatric nurses, the attitudes and perspectives of psychiatric nurses on euthanasia based on UMS has not yet been explored. Our study aimed to explore the attitudes and perspectives of psychiatric nurses in residential psychiatric settings. We also assessed to which extent nurses are confronted with and engaged in EOL questions from patients and whether they perceived gaps in knowledge and skills related to this complex topic.

## Materials and Methods

### Design

A multicenter study with a quantitative descriptive design. The questionnaire was developed from existing scales and instruments used in the field of nursing. The questionnaires considered for inclusion were based on a broad literature review (PubMed, CINAHL, Cochrane and Psychoinfo) for scales or questionnaires which evaluated attitudes of nurses towards euthanasia regardless of the type of suffering (both physical and mental). Studies not specifically targeting nurses (e.g. only physicians) were excluded. A total of 6 studies were identified [11–16] and underwent a methodological quality assessment (S1 File)

No specific questionnaires were available for nurses in psychiatric settings; therefore certain questions were adapted to meet the unique needs of these services. Since none of the previous studies dealt with euthanasia based on UMS, 9 items were developed based on available literature on the topic. The aim was to have neutral questions or statements that did not contain double meanings.

Answer categories were as wide as possible in order to capture all alternatives from respondents. Some questions were on a 5 point Likert scale (fully agree to fully disagree). Other questions were dichotomous requiring a yes or no response. The survey also had additional free text at the end for personal notes or comments (S2 File). Each survey was only obtained after obtaining informed consent from the participant.

### Population and setting

Based on a non-probabilistic sampling, 20 regional and university psychiatric hospitals (Dutch speaking part of Belgium) were selected for participation in the study. Minimally 1 and maximally 4 hospitals were selected per province, based regional spread in the catchment areas. Selection was performed at random on the basis of the national hospital list. A contact person was identified and contacted by a researcher (LVB). Participation to the study was proposed to the local ethic committee of each hospital.

We included the following wards: acute admission wards, wards for treatment and rehabilitation and partial treatment wards in hospital settings for adult patients. Excluded were chronic board facilities, sheltered housing projects, exclusively ambulatory treatment settings, and wards for children and adolescents were also excluded because a legal framework for youngsters was not available at that time. All psychiatric nurses of the selected wards were asked to participate (N = 849).

### Procedures

Questionnaires were distributed to the participants electronically or in paper version. Paper versions were collected in closed boxes on the ward. The paper version was distributed on each ward by a researcher (LVB). The researcher also explained the nature and purpose of the study through meetings or individual contacts with head nurses. Electronically filled out questionnaires were centrally collected by a researcher (LVB). Paper versions were collected in closed boxes on the ward. The questionnaires were available for 31 days.

### Statistical analysis

Descriptive statistics were computed for basic demographic and clinical variables as well as the answers on the survey questions. Associations between two categorical variables were evaluated by means of a chi-square test, whereas associations between two continuous variables or a continuous variable and a categorical variable were tested with ANOVA. The item probing respondents agreement with euthanasia based on UMS was recorded to 2 levels (completely agree and agree were combined in 1 category, the 3 other response options were grouped in a 2^nd^ category). Logistic regression was used to assess the influence on these dichotomous variables of gender, diploma, work experience, type of ward, ward population, interpretation of euthanasia either as a wish to live or as a wish to die, and whether one thought that discussing euthanasia would either increase or decrease the patients’ death wish. Alfa level was set at 0.05, and all tests were two-sided. Statistical analysis was performed with SAS (Statistical Analysis System, Cary, North Carolina, USA).

### Ethical considerations

The study was approved by the central ethics committee of the KU Leuven, the local ethic committees of the hospitals in Ieper, Brugge, Duffel, Rekem and Kortenberg.

## Results

In total, 3 university and 8 regional psychiatric hospitals agreed to participate (11 institutions). At least 1 hospital per Flemish province participated. Based on the potential target population of nurses, 849 questionnaires were distributed. After the 31 day recruitment period, 627 surveys were returned (598 on paper and only 29 electronically). Nearly all hospitals made use of the paper version, only one hospital used the electronic version. The global response rate was 73%. Three questionnaires were excluded from analysis for incompleteness (<50% completed). With the exception of 1 hospital (response rate of 40%), the response rate varied between 59% and 100%. The hospital with the lowest response rate was a facility which opted for exclusive electronic data capture.

Characteristics of the participants are shown in Table 1. The largest group of nurses worked on wards where different psychiatric disorders were being treated. The 2^nd^ largest group worked on wards with patients suffering mainly from psychotic disorders. The majority of respondents were 35 years or older. More than half worked for more than 10 years. Most of the participants were female.

**Table 1:** Demographic data participants.

Variable	Frequency n/(%)
**Provence**	**N = 624**
Antwerpen	115(18)
Limburg	60 (10)
Oost-Vlaanderen	93 (15)
Vlaams-Brabant	124 (20)
West-Vlaanderen	232 (37)
**Age range participant**	**N = 622**
20–24	68 (11)
25–34	187 (30)
35–44	151 (24)
45–54	143 (23)
>55	73 (12)
**Sex**	**N = 623**
Male	166 (27)
Female	457 (73)
**Diploma**	**N = 620**
Higher professional education	262 (42)
Bachelor	314 (51)
Bachelor after bachelor	31 (5)
Master	13 (2)
**Work experience of the participant**	**N = 622**
0–2 years	76 (12)
2–5 years	100 (16)
5–10 years	88 (14)
>10 years	358 (58)
**Type of ward**	**N = 598**
Acute	327 (55)
Rehabilitation or long-term treatment ward	271 (45)
**Patient population of the ward**	**N = 622**
Mixed	315 (51)
Personality disorder	54 (9)
Mood disorder	38 (6)
Schizophrenia or psychosis	146 (23)
Addiction	69 (11)

### Attitudes of nurses towards euthanasia based on UMS

The largest number of participants did not object euthanasia based on UMS. Only a small minority stated that euthanasia should be restricted only to physical suffering, while 19 participants found euthanasia ethically unacceptable.

Over two-thirds reported that palliative care and euthanasia are mutually exclusive, while two small groups either did not agree or had a mixed opinion.

A minority of nurses considered that all psychiatric patients lack all decision making capacity, while the largest group had a neutral point of view and according to a smaller group mentally ill patients have capacity of will to request euthanasia (Table 2).

**Table 2:** Knowledge and attitudes the law on euthanasia.

Statement	Total (n = 624)	Agree N (%)	Not agree N (%)	Neutral N (%)
1. Based on the current law on euthanasia (2002), euthanasia is being performed too quickly or easily (both in somatic medicine and psychiatry).	622	16 (2.5)	525 (84)	81 (13)
2. Euthanasia should be restricted to unbearable somatic suffering	620	12 (2)	574 (93)	34 (5)
3. From my personal ethics perspective euthanasia should never be performed.	623	19 (3)	566 (91)	38 (6)
4. I agree with the current legislation allowing euthanasia based on UMS.	623	536 (86)	24 (4)	63 (10)
5. Euthanasia and palliative medicine are mutually exclusive.	601	76 (13)	424 (70)	101 (17)
6. A psychiatric patient has decision making capacity.	615	280 (46)	34 (5)	301 (49)
7. Many patients think that with the current legislation they have a right to euthanasia. Do you agree that the physician should always/ is morally obligated to agree with the request for euthanasia.	616	89 (14)	388 (63)	139 (23)

### Factors that influence nurses attitudes

The null model of the logistic regression was rejected (χ^2^(17) = 37.71, p = 0.0027) indicating that some predictors were significantly associated with agreement to euthanasia based on UMS. Both the type of ward (χ^2^(1) = 10.96, p = 0.0009), and the diagnosis of patients (χ^2^(4) = 10.85, p = 0.0283), were significantly associated with agreeing to euthanasia. Specifically, nurses working in acute settings were more likely to agree with the patients’ request for euthanasia compared to respondents working in longer term treatment settings (OR = 2.5; CI:1.5–4.3). Nurses working with either psychotic patients (OR = 3.9; CI: 1.6–9.3), patients with personality disorders (OR = 3.7; CI:1.2–12.1), or divers patient population (OR = 2.4, CI: 1.2–5) were more likely to agree with euthanasia based on UMS in comparison to nurses working with patients with addiction.

The complete model is shown in Table 3. There were no significant associations with sex, diploma, years of work experience, judgement on capacity of will nor suicide related items.

**Table 3:** Logistic regression: influence on attitude towards euthanasia base on UMS.

Variable	Χ^2^	Df	Pr>Chi^2^	Odds ratio (OR)	Confidence interval (CI)
Sex	0.9573.	1	0.3279		
Diploma	1.0887	3	0.7998		
Work experience	4.1067	3	0.2502		
Population	10.8497	4	0.0283	**Mixed vs. Addiction** 2.4	**Mixed vs. Addiction** 1.2–5.0
				**Psychosis vs. Addiction** 3.9	**Psychosis vs. Addiction** 1.6–9.3
				**Personality disorder vs. Addiction** 3.7	**Personality disorder vs. Addiction** 1.2–12.1
				**Mood disorder vs. Addiction** 3.9	**Mood disorder vs. Addiction** 0.8–20.1
Type of ward	10.9641	1	0,0009	**Acute vs. Rehabilitation** 2.5	**Acute vs. Rehabilitation** 1.5–4.3
Capacity of will	0.0360	1	0.8495		
Discuss euthanasia UMS* = increases death wish	3.6283	1	0.0568		
Discuss euthanasia UMS = decrease death wish	0.0008	1	0,9773		
Euthanasia = request to cope/live	1.7913	1	0.1808		
Euthanasia = suicide	3.4508	1	0,0632		

### Confrontation with euthanasia requests in daily clinical practice

More than half of all participating nurses have been directly confronted with a request for euthanasia by a psychiatric patient, and even more nurses had knowledge about a demand through a colleague (Table 4). The frequency varied from once (53%) to more than 3 times (32%). A smaller number indicated that euthanasia had been performed at least once in the hospital where they worked.

**Table 4:** Confrontation with euthanasia request based on UMS

	Yes N (%)	No N (%)
Direct confrontation with euthanasia request	329 (53)	293 (47)
Via information of another mental health worker	427 (69)	191 (31)
Was euthanasia performed in the psychiatric facility	82 (13)	536 (87)

The largest proportion of demands was in patients between 40 and 60 years old. The number of demands was equally distributed across diagnostic groups, including psychotic disorders, personality disorders and mood disorders. Multiple diagnoses were present in 17% of all cases.

When confronted with a request based on UMS, the question was discussed individually with the patient in 72% of all cases, but most of the time it was also discussed in the multidisciplinary teams and referred to the treating psychiatrist. In only 7% of all cases the request was ignored.

According to the participants view 36 patients died with euthanasia. Euthanasia had been performed in all institutions except for one (a small rehabilitation unit), and in 2 of 3 cases the main diagnosis was a personality disorder(Table 5).

**Table 5:** Direct confrontation with euthanasia request based on UMS.

	N(%)
Frequency	**327 (38,5)**
Once	99 (30)
Twice	90 (28)
3-times	32 (10)
>3 times	106 (32)
Patient characteristics*	
Age	**323 (38)**
<18	4 (1)
18–30	78 (24)
30–40	82 (25)
40–60	131 (41)
>60	28 (9)
Sex	**316 (37,2)**
Male	124 (39)
Female	192 (61)
Diagnosis	**319 (37,5)**
Personality disorder	73 (23)
Psychotic disorder	87 (27)
Mood disorder **	77 (24)
Other ***	82 (26)
Approach to request	
Discussed with patient	**325 (38,2)**
Yes	233 (72)
No	92 (28)
Ignore	**312 (36,7)**
Yes	21 (7)
No	291 (93)
Referred to psychiatrist	**320 (37,6)**
Yes	290 (91)
No	30 (9)
Discussed in team	**304 (35,8)**
Yes	289 (95)
No	15 (5)
Course	
Was euthanasia performed	**317 (37,3)**
Yes	36 (11)
No	281 (89)

* N nurses who gave a description of patient characteristics.

**Major depression and bipolar disorder

***Huntington, dementia, combination with somatic disease, eating disorder, substance abuse, combination of different diagnosis 53 (16)

### Competencies of psychiatric nurses

The opinion of psychiatric nurses about their role and competences are shown in Table 6. More over 80% of psychiatric nurses judged that they have the ability and should be allowed to discuss a euthanasia request with the patient. They are convinced that these discussions should not only be held by the physician. Most of the participants consider input of psychiatric nurses of crucial importance in the evaluation of euthanasia requests.

**Table 6:** Role and competencies psychiatric nurses.

Role of psychiatric nurse	N(%)	Agree	Not agree
**Question**	**624**		
1. Should discussions with the patient about euthanasia solely be done by the psychiatrist?	618	24 (4)	548 (89)
2. Should and can a psychiatric nurse discuss euthanasia with the patient?	619	547 (88)	13 (2)
3. Is the input of the psychiatric nurse crucial in the assessment and evaluation of a request for euthanasia?	621	436 (70)	48 (8)
**Competence development**		**Yes**	**No**
4. Do you have sufficient knowledge, information and skills to deal with a request for euthanasia by a patient?	613	179 (29)	434 (71)
5. Has the topic been covered in your psychiatric nursing training?	619	185 (30)	160 (26) **NA** * 274 (44)
6. Is it important that the topic is covered in the training for mental health nurses?	622	618 (99)	4 (1)
**Relevance of the topic**		**Yes**	**No**
7. Did you find participation in this study useful?	606	578 (95)	28 (5)
8. Do you find the topic relevant for your daily clinical practice?	600	485 (81)	115 (19)

* NA. Not applicable because no law on euthanasia before 2002 274()

However, the majority felt that they lacked information, knowledge and/or skills to adequately deal with a euthanasia request by psychiatric patients. The topic was not adequately addressed in the professional or academic training. The majority of participants found that the topic of euthanasia based on UMS should be an integral part of the training curriculum. Nearly all participants appreciated participation in the study and found the topic of clinical relevance.

## Discussion

To our knowledge this study is the first to address perspective and attitudes of psychiatric nurses towards euthanasia based on UMS by psychiatric patients.

Within general medicine a request for euthanasia is a stepwise process that requires specific competences of all involved health care workers [17]. In our study, a majority of psychiatric nurses indicate that they lack information and skills for dealing with a request made by psychiatric patients. Nevertheless, requests for euthanasia are common and increasing annually. More than half (56%) of all participants were confronted with a euthanasia request.

An important question is how nurses can respond to these requests in an ethically responsible manner. At first, they and all involved team members should critically examine their own general attitudes towards euthanasia [18]. A process of ethical decision making is not only a cognitive process, but is also influenced by personal and contextual factors [19]. Requests of euthanasia should be met in a multidisciplinary approach where a physician has to decide over various aspects: the decision making capacity of the patient, legal aspects of the requests and his/her own attitude towards euthanasia.

On the individual patient level, decision making capacity has to be considered and formally assessed. A small minority of nurses judged that patients have intact capacity of will. But there was no significant relationship between not agreeing with euthanasia and judging that there was incapacity of will in patients. A frequent comment of respondents was the lack of reliable instruments to evaluate capacity of will. A general statement that psychiatric patients have incapacity of will can be rejected and should be replaced by a functional assessment of how the current mental state has an impact on relevant functions of will [20]. There is consensus on capacity of will: a) capacity of will is intact until proven not to be the case otherwise [20; 21]; b) capacity of will can vary over time and with specific situations or choices, requiring repeated evaluations over time; and c) capacity of will is task specific and the assessment should target the decision making process and not the decision as such [21; 22].

In the decision making process about euthanasia based on UMS, the unbearability of mental suffering is one of the more complicated aspects. What is mental suffering and how unbearable is it? UMS is what the patient says it is and there for subjective [2]. If mental suffering is a symptom of the psychiatric illness, then it becomes treatable and the unbearability maybe fades away. Some requests can be a cry for help and if they are well assessed a possible therapeutic instrument [23]. If dealing with this requests works therapeutic maybe nurses could have an important intervening role. Nurse have a unique position between doctors and patients. The majority (93%) of nurses did not agree with a restriction of euthanasia only for unbearable physical suffering. A potential relationship to active or passive suicidal ideation had no effect whether nurses agree or disagree with euthanasia, which highlights that nurses recognize and acknowledge UMS.

When a request for euthanasia has been denied sometimes risk of suicide increases and patients obstinate by looking for other well-willing physicians [6; 24]. The pressure on the deciding physician can be countered for by multidisciplinary approach. Dealing with such complex requests could impact the ethical attitude of the physician regarding euthanasia requests. The law for euthanasia is not a right that obliges a physician to administer euthanasia in general. There for it is important that a physician knows his own point of view and communicates this openly towards his team and the patient. Nurses can have a supportive role towards the physician in dealing with and communicating about the request because of their unique position towards patients. The specific relationship between nurses and patients based on trust and the empathic attitude of nurses could be a potential reason why nurses have a sensitive view on the mental suffering of their patients. Therefore, nurses may be more open to consider and accept the patients’ will.

More than 80% of all participating nurses did not object euthanasia based on UMS. The amount to which nurses agree was influenced by two confounders: the type of ward and the diagnosis of the patient. Nurses working on rehabilitation wards tended to disagree more with euthanasia based on UMS than nurses working on acute wards. A possible explanation could be that rehabilitation models focus on recovery are more likely to install hope in nurses that patients can improve, regain autonomy and purpose in life. A long term care for patients may also lead to closer therapeutic relationships which could influence a point of view towards euthanasia. Another explanation could be that nurses working on acute wards, see patients when the illness is most severe and extreme. Effects of treatment are more visible and rewarding on such wards. But what happens to patients who get admitted several times on yearly base? Maybe that could be why nurses on acute wards look different to euthanasia based on UMS.

Nurses tend to agree more to requests made by psychotic patients, patients with mood and/or personality disorders in comparison to patients with addiction. It seems somewhat strange that nurse tend to disagree more to requests made by patients with an addiction. When patients have a co-occurring psychiatric disorder with an alcohol dependence the chance to improve patients functioning only increases when treating both disorders simultaneously. Without complete management of all existing problems full recovery may be impossible [25]. From this perspective the trend that nurses having in disagreeing to requests made by patients with an addiction is not so unexpected. From our results it is not clear if patients with an addiction had a co-occurring psychiatric disorder. Most wards in Belgian mental health who treat addictions, primarily work with the problem of the addiction, therefore are not able to decide whether a patients request is legally solid. Is the request then in such a state of constant and unbearable mental suffering, which cannot be alleviated? Maybe treatment fails by not dealing with both occurring disorders at the same time.

Another important finding is that 70% of all participated nurses indicate that euthanasia and palliative care are closely intertwined and complement one another [8; 26; 27]. According to the World Health Organization, palliative care is an approach that improves the quality of life of patients and their families facing the problem associated with life-threatening illness, through the prevention and relief of suffering by means of early identification and impeccable assessment and treatment of pain and other problems, physical, psychosocial and spiritual [28]. Psychiatric patients are confronted with high rates of somatic co-morbidities [29]. Therefore, it can be assumed that palliative care should not only be limited to terminally ill patients but should also preventively address somatic disorders/complications in the future [30; 31]. Offering information about palliative options and comfort care could be helpful in clarifying the request for help, differentiating between a wish for suicide and a euthanasia request [6; 32]. According to Sweers et al. psychiatric nurses have a unique opportunity, due to the close and frequent contact with patients, to explore the options and needs for palliative care [23] and in dealing with requests of euthanasia. We found that 88% thinks that a psychiatric nurse should have the possibility to discuss the request with the patient. Involving psychiatric nurses in assessing and evaluating a request for euthanasia is for more than half of all participated nurses crucial. Also in general health care nurses want to be actively involved in the decision making process [33].

The high figure of agreement and the wish to be involved in the decision making process of requests for euthanasia based on UMS is remarkable. Worrisome are the reported lack of knowledge, information and skills on this complex subject. Almost half of all participating nurses found that this topic is not integrated in their training for psychiatric nursing and yet 99% think it is important to integrate this topic. Prior research has already indicated the general need of developing a personal moral and professional attitude towards euthanasia early in nursing training [33]. Psychiatric nurses need to develop skills in how to discuss end-of-life concerns and needs with psychiatric patients in general, but also in acute situations. When a psychiatric patient is an acute phase of the illness, these end-of-life needs maybe different and very specific.

The results of this study have to be interpreted with caution. Although the high response rate suggests that the results could be representative, the internal validity can be questioned because of the non-random selection of hospitals. Moreover, the questionnaire has not been validated and was developed from existing questionnaires in somatic settings. Different nurses could have given information on the same patient, so that we do not have sound prevalence data per diagnostic group (Table 5). Religion which might be an important factor in attitudes towards euthanasia, was not evaluated [33]. The hospital with the lowest response rate (41.8%) was the only facility which opted for exclusive electronic data capture. Future research should explore the incidence and content evaluation of euthanasia requests based on UMS. Another focus could be on specific age, belief systems or diagnostical groups. Qualitative research could give more insight into the intensity and better description and understanding of UMS [34].

Data of this kind of research can be useful in handling individuals with euthanasia requests. Lastly, further research is needed on adapted guidelines for a possible integration of euthanasia and palliative care in psychiatric settings.

## Conclusion

Euthanasia remains an ethically sensitive topic. Demands based on UMS add to the complexity of the issue. The results of our study highlight the need for adequate and ethically evidence based care. More than half of the nurses have already been confronted with a request for euthanasia by a psychiatric patient. There is a clear need for more training on the topic. An integration of palliative care and end-of-life decisions which includes euthanasia should be introduced in psychiatric care facilities. Additional research is needed both on perspectives of patients and caregivers, as well as other mental health professionals.

## Supporting Information

S1 File Survey A. English translation of the survey.
(DOCX)

S2 File Survey B. Survey in Dutch.
(PDF)
