# Supplementary material for: Attitudes of Psychiatric Nurses about the Request for Euthanasia on the Basis of Unbearable Mental Suffering(UMS)
Source: PLoS One. 2015 Dec 23;10(12):e0144749. doi: 10.1371/journal.pone.0144749 (PMC4689522; doi:10.1371/journal.pone.0144749)
Supplement: S1 File — English translation of the survey. (DOCX) [file pone.0144749.s001.docx]

**Survey**

**Demographic data: (mark the square that applies to you)**

| Age | - 20-24 - 25-34 - 35-44 - 45-54 - >55 |
| --- | --- |
| Gender | - Male - Female |
| Degree  (Highest completed degree) | - Graduated nurse (A2) - Bachelor in nursing (A1) - Bachelor after bachelor (mental health or other) - Master in nursing and midwifery |
| Patient population  (main diagnosis) | - Mood disorders (depression, bipolar disorder and/ or anxiety disorder) - Psychosis (schizophrenia and/ or other psychotic disorders) - Personality disorders - Addictive disorders - Eating disorders - Pervasive development disorders for adults (autism spectrum disorder) - Attention deficit hyperactivity disorder (ADHD adults) - Neurotic disorders (obsessive compulsive disorder) - Diverse (= different diagnoses on 1 ward) |
| Work experience  (…years) | - 0-2 - 2-5 - 5-10 - >10 |
| Type ward | - Day centre - Acute - Observational/ diagnosis - Resocialisation/ long term care |

**PART 1: Point of view concerning the law for euthanasia.**

| **** Euthanasia:*** *intentionally terminating life by some other than the person concerned, at the latter’s request.*  **** Law for euthanasia 28 may 2002:*** *The physician who performs euthanasia commits no criminal offence when he/she ensures that: 1) the patient has attained the age of majority or is an emancipated minor, and is legally competent and conscious at the moment of making the request, 2) the request is voluntary, well-considered and repeated, and is not the result of any external pressure, 3) the patient is in a medically futile condition of constant and unbearable physical or mental suffering that cannot be alleviated, resulting from a serious and incurable disorder caused by illness or accident.* |
| --- |

| 1.A: Based on the euthanasia law of 28 may 2002, I think that euthanasia in clinical practice has been applied too quick and without caution ( in general and mental health). | - Totally agree - Agree - Neither agree nor disagree - Disagree - Totally disagree |
| --- | --- |
| 1.B: The law for euthanasia should only be applied to physical suffering. | - Totally agree - Agree - Neither agree nor disagree - Disagree - Totally disagree |
| 1.C: I have ethical concerns against euthanasia. | - Totally agree - Agree - Neither agree nor disagree - Disagree - Totally disagree |
| 1.D: In our current legal framework concerning euthanasia requesting euthanasia based on unbearable mental suffering is possible. I agree with this statement (= I don’t have any problem with this statement). | - Totally agree - Agree - Neither agree nor disagree - Disagree - Totally disagree |
| 1.E: Euthanasia and palliative care exclude one another. | - Totally agree - Agree - Neither agree nor disagree - Disagree - Totally disagree |
| 1.F: I believe that a psychiatric patient has decision making capacity. | - Totally agree - Agree - Neither agree nor disagree - Disagree - Totally disagree |
| 1.G Since euthanasia has been legalized in 2002, patients (with unbearable physical and mental suffering) presume that it is a right to receive euthanasia. According to the patient, doctors are morally obliged to agree with a request to euthanasia. Do you agree with this presumption? | - Totally agree - Agree - Neither agree nor disagree - Disagree - Totally disagree |

**PART 2: Frequency of euthanasia requests based on unbearable mental suffering.**

| 2.A: Were you directly confronted with a patient who had a request for euthanasia based on unbearable mental suffering ? | - Yes - No (Go to 2B) |
| --- | --- |

**If Yes:** (**Comment:** Frequency >1: **you can only describe one patient. Choose the most current situation.**

| 2.1 Frequency |  |
| --- | --- |
| How many times has this occurred? | - Once - 2 times - 3 times - > 3 times |
| 2.2 Characteristics of the patient |  |
| Age of the patient? | - <18 - 18-30 - 30-40 - 40-60 - >60 |
| Gender of the patient? | - Male - Female |
| Diagnosis of the patient? (main complaint) | - Schizophrenia (spectrum: e.g., schizo-affective and others.) - Depression (major) - Depression (dysthymia) - Anxiety disorder - Eating disorder - Personality disorder - Bipolar disorder - Addictive disorder - Obsessive compulsive disorder - Pervasive development disorder - Autism spectrum disorder (adults) - Other (specify: _ _ _ _ _ _ _ _ _ _ _ _ _ _ _ ) |
| 2.3 Approach |  |
| Did you discuss the request for euthanasia with the patient? | - Yes - No |
| Did you ignore the request?  (this also includes that the request for euthanasia was considered as a suicide wish ). | - Yes - No |
| Did you referred the patient to a psychiatrist?  (you did not intervene yourself). | - Yes - No |
| Did you discuss the request with the team? | - Yes - No |
| 2.4 Execution |  |
| Was the request for euthanasia carried out? | - Yes - No |

| 2.B: Has there been a (in)direct request for euthanasia from a psychiatric patient who is/was admitted in your hospital? | - Yes - No |
| --- | --- |
| 2.C: Has there been an execution of euthanasia in the hospital you are working in? | - Yes - No |

**PART 3: Nurses attitude and point of view:**

| 3.A: Allowing to talk about euthanasia with the psychiatric patient may increase their wish to die | - Totally agree - Agree - Neither agree nor disagree - Disagree - Totally disagree |
| --- | --- |
| 3.B: Allowing to talk about euthanasia with the psychiatric patient may decrease their wish to die. | - Totally agree - Agree - Neither agree nor disagree - Disagree - Totally disagree |
| 3.C: The request for euthanasia based on unbearable mental suffering is a cry for help. | - Totally agree - Agree - Neither agree nor disagree - Disagree - Totally disagree |
| 3.D: The request for euthanasia based on unbearable mental suffering can be considered as suicidal thoughts (passive or active). In such cases, preventive measures concerning suicide should be a priority. | - Totally agree - Agree - Neither agree nor disagree - Disagree - Totally disagree |

**PART 4: The nurses position (1):**

| 4.A: Discussing euthanasia with the patient is only a matter for the psychiatrist. | - Totally agree - Agree - Neither agree nor disagree - Disagree - Totally disagree |
| --- | --- |
| 4.B: A psychiatric nurse has the ability to discuss the euthanasia request with the patient. | - Totally agree - Agree - Neither agree nor disagree - Disagree - Totally disagree |
| 4.C: In the assessment of euthanasia requests involving the responsible caretaker (nurse) is crucial. | - Totally agree - Agree - Neither agree nor disagree - Disagree - Totally disagree |

**PART 5: The nurses position (2):**

| 5.A: When you would be confronted with a euthanasia request, are you convinced that you have enough knowledge, skills and information to deal with this request? | - Yes - No |
| --- | --- |
| 5.B: Has the topic of euthanasia been addressed in your training in the context of mental health? | - Yes - No - Not applicable (law for euthanasia   < 2002 not yet approved) |
| 5.C: Do you think it is important that the topic of euthanasia will be integrated in the training for future nurses (especially for psychiatric nurses)? | - Yes - No |

**Opinion about survey:**

| Did you find participating in this study useful? | - Yes - No |
| --- | --- |
| Do you think this study is clinically relevant? | - Yes - No |
| Comments: |  |

**Thank you for participating.**
