# Supplementary material for: Attitudes of Psychiatric Nurses about the Request for Euthanasia on the Basis of Unbearable Mental Suffering(UMS)
Source: PLoS One. 2015 Dec 23;10(12):e0144749. doi: 10.1371/journal.pone.0144749 (PMC4689522; doi:10.1371/journal.pone.0144749)
Supplement: S2 File — Survey in Dutch. (PDF) [file pone.0144749.s002.pdf]

Informatie en toestemmingsformulier

Attitude van psychiatrisch verpleegkundigen ten aanzien van het verzoek tot euthanasie op basis van ondraaglijk psychisch/psychiatrisch lijden.

Opdrachtgever(s): Professor Dr. Marc De Hert, Onderzoeks- en Leifnurse Mevr. Kim Sweers, Masterstudent verpleegkunde en vroedkunde Liesbet Van Bos.

Geachte,

Aan de hand van dit informatie- en toestemmingsformulier wens ik u voldoende informatie aan te reiken betreffende deelname aan wetenschappelijk onderzoek.

Inleiding:
Er wordt u gevraagd om deel te nemen aan een wetenschappelijk onderzoek omtrent: *Attitude van psychiatrisch verpleegkundigen ten aanzien van het verzoek tot euthanasie op basis van ondraaglijk psychisch/psychiatrisch lijden.* In de tekst hieronder wordt u uitgelegd wat de bedoeling van deze studie is en wat een eventuele deelname voor u betekent.
Vooraleer te beslissen al dan niet deel te nemen aan dit onderzoek, vragen we u deze informatie aandachtig te lezen.

Deze studie werd beoordeeld en goedgekeurd door de Commissie voor Medische Ethiek van de UZ/K.U.Leuven. U dient deze goedkeuring niet te beschouwen als een aansporing tot deelname aan deze studie.

Doel van het onderzoek:
Huidige berichtgeving in de media en cijfermatige onderzoekgegevens werpen een blik op de toenemende vraag van psychiatrische patiënten naar euthanasie op basis van psychisch/psychiatrisch ondraaglijk lijden. Het opzet van huidig onderzoek is bedoeld om uw visie als psychiatrisch verpleegkundige m.b.t. euthanasieverzoek van de psychiatrische patiënt in kaart te brengen.

Door middel van uw visie en standpunten kan een vergelijking gemaakt worden met onderzoeksgegevens over attitudes betreffende palliatieve zorg en euthanasie bij somatisch verpleegkundigen. Op basis van onderzoeksresultaten zou er eventueel een hypothese kunnen gevormd worden over mogelijk 'nieuwe' zorgnoden voor de psychiatrische patiënt. Tevens zou op basis van dit onderzoek al dan niet mogelijke hiaten in de psychiatrisch verpleegkundige zorg geformuleerd kunnen worden, met als doel hier in de toekomst op in te spelen.

Praktische uitvoering van het onderzoek:
Het onderzoek vindt plaats op de afdeling waar u bent tewerkgesteld tijdens de arbeidsuren. U hoeft zich niet te verplaatsen. Een vragenlijst wordt gedurende 31 dagen elektronisch of schriftelijk op uw afdeling ter beschikking gesteld. Na het vervolledigen van de vragenlijst kan u deze, indien elektronisch, opslaan en doormailen naar:
Liesbet.vanbos@student.kuleuven.be (instructies voor doorsturen van vragenlijst worden vermeld in vragenlijst).

Indien schriftelijk, kan u deze in een gesloten box deponeren welke door de onderzoekster (masterstudent) persoonlijk wordt opgehaald.

Mogelijke risico's:
Mogelijk kan het thema dat aan bod komt in de vragenlijst voor u persoonlijk bepaalde ervaringen of gevoelens oproepen. Indien dit het geval zou zijn, wordt aangeraden hierover te ventileren op een manier die voor u de meeste ondersteuning kan bieden. In samenspraak met uw ziekenhuis kan u ook bij bepaalde vragen of emoties terecht bij de psycholoog van uw afdeling, die zo nodig verdere stappen kan ondernemen.
Mogelijke voordelen:
Uw medewerking aan wetenschappelijk onderzoek kan een bijdrage leveren aan een meer gerichte zorg op maat van de psychiatrische patiënt en levert u een persoonlijke inbreng tot verdere optimalisatie van het wetenschappelijk verpleegkundig vakgebied.
Kosten en vergoeding:
Deelname aan dit onderzoek zal geen kosten met zich meebrengen.
Verzekering:
Indien u schade oploopt ten gevolge van deelname aan deze studie, zal de schade vergoed worden conform de Belgische wet aangaande experimenten op de menselijke persoon van 7 mei 2004. Volgens deze wet is de opdrachtgever van het onderzoek, (Professor Dr. Marc De Hert), zelf foutloos aansprakelijk voor alle schade die de deelnemer en/of zijn rechthebbenden oplopen en die rechtstreeks dan wel onrechtstreeks verband houdt met de proef. De opdrachtgever heeft een verzekering afgesloten die deze aansprakelijkheid dekt.
Vrijwillige deelname:
U bent volledig vrij om te beslissen al dan niet aan dit onderzoek deel te nemen. Indien u beslist om niet deel te nemen zal u hierdoor op geen enkele manier nadeel ondervinden. Bovendien heeft u het recht om op ieder ogenblik de deelname aan de studie stop te zetten, zonder hiervoor een verklaring te moeten geven.
Vertrouwelijkheid:
De Belgische Wet van 11 december 1998 betreffende de bescherming van het privéleven wordt gerespecteerd bij het uitvoeren van dit onderzoek. Alle persoonlijke informatie die tijdens deze studie wordt verzameld is bijgevolg strikt vertrouwelijk. Zij wordt enkel aangewend voor het doeleinde van deze studie. Alle persoonlijke informatie wordt gecodeerd. (*In de code mogen geen data voorkomen die kunnen leiden tot identificatie van de proefpersoon bv combinatie van geboortedatum en initialen is niet toegelaten)*. Uw identiteit wordt op geen enkele manier kenbaar gemaakt aan onbevoegde derden. Bij eventuele publicatie van de gegevens over deze studie zal nooit enige informatie bekend gemaakt worden die uw identiteit kan kenbaar maken.
Opmerkingen:
Bij eventuele vragen in verband met deze studie kan u zich steeds richten tot uw onderzoekarts en hoofdgeneesheer Professor Dr. Marc De Hert, onderzoekster en LEIFnurse Mevr. Kim Sweers en masterstudent Van Bos Liesbet:
Email: Liesbet.vanbos@student.kuleuven.be
GSM: 0496/117.178

Toestemmingsformulier

Door het invullen van de vragenlijst verklaart u bovenstaande informatie zorgvuldig te hebben nagelezen en neemt u vrijwillig deel aan deze studie. U hebt het recht om op ieder ogenblik zonder enige gevolgen uw deelname stop te zetten zonder dat u hiervoor een reden dient op te geven.

Vragenlijst

Demografische gegevens: (aankruisen wat voor u van toepassing is)

**Leeftijd**	❑ 20-24<br>❑ 25-34<br>❑ 35-44<br>❑ 45-54<br>❑ >55
**Geslacht**	❑ Man<br>❑ Vrouw
**Diploma**<br>**(hoogst behaalde)**	❑ Hoger beroepsonderwijs/ gebrevetteerde verpleegkundige (A2)<br>❑ Bachelor verpleegkunde/ gediplomeerde verpleegkundige (A1)<br>❑ Bachelor na bachelor (geestelijke gezondheidszorg of andere)<br>❑ Master verpleegkunde en vroedkunde
**Patiëntenpopulatie**<br>**(meest voorkomend op afdeling, primaire hoofddiagnose)**	❑ Stemmingsstoornis (depressie, bipolaire stoornis en/ of angststoornis)<br>❑ Psychose (schizofrenie en/ of andere aandoeningen m.b.t. psychose)<br>❑ Persoonlijkheidsstoornis<br>❑ Verslavingsproblematiek<br>❑ Eetstoornis<br>❑ Pervasieve ontwikkelingsstoornis bij volwassenen (autisme spectrumstoornis)<br>❑ Attention deficit hyperactivity disorder (ADHD volwassenen)<br>❑ Neurotische stoornissen (obsessief compulsieve stoornis)<br>❑ Divers (= verschillende diagnosen op afdeling)
**Werkervaring**<br>**(Aantal jaren)**	❑ 0-2<br>❑ 2-5<br>❑ 5-10<br>❑ >10
**Aard afdeling**	❑ Dagcentrum<br>❑ Acuut (opname)<br>❑ Observatie/ diagnose<br>❑ Resocialisatie/ langdurige zorg

REEKS 1: Vragen met betrekking tot visie wetgeving euthanasie:

*** Euthanasie:** *het opzettelijk levensbeëindigend handelen door een andere dan de betrokkene, op diens verzoek.*

*** Wetgeving euthanasie 28 mei 2002:** *De patiënt die om euthanasie verzoekt dient meerderjarig en bewust te zijn op het ogenblik van het verzoek (mogelijke aanpassing: wordt voor de eerste keer besproken in senaat op 20/02/2013 waardoor de wet minderjarigen wel toelaat. Is echter nu nog niet van toepassing). Het verzoek dient vrijwillig, overwogen en herhaald te zijn en niet tot stand gekomen als gevolg van enige externe druk. De patiënt bevindt zich in een medisch uitzichtloze toestand van aanhoudend en ondraaglijk fysisch of psychisch lijden dat niet gelenigd kan worden, en dat het gevolg is van een ernstige ongeneeslijke, door ongeval of ziekte veroorzaakte aandoening.*

**1.A:** Op basis van de huidige euthanasiewetgeving (28 mei, 2002) wordt euthanasie volgens mij te snel en/of te gemakkelijk toegepast in de praktijk (zowel somatische als psychiatrische zorg).	❑ Helemaal akkoord<br>❑ Akkoord<br>❑ Niet/ wel akkoord<br>❑ Niet akkoord<br>❑ Helemaal niet akkoord
**1.B:** De euthanasiewetgeving dient enkel behouden te worden tot fysisch lijden.	❑ Helemaal akkoord<br>❑ Akkoord<br>❑ Niet/ wel akkoord<br>❑ Niet akkoord<br>❑ Helemaal niet akkoord
**1.C:** Voor mij persoonlijk is euthanasie nooit ethisch verantwoord.	❑ Helemaal akkoord<br>❑ Akkoord<br>❑ Niet/ wel akkoord<br>❑ Niet akkoord<br>❑ Helemaal niet akkoord
**1.D:** Op basis van de huidige euthanasiewetgeving is euthanasie op basis van psychisch ondraaglijk lijden mogelijk (zie wetgeving). Ik ga hiermee akkoord (= ik heb hier geen probleem mee).	❑ Helemaal akkoord<br>❑ Akkoord<br>❑ Niet/ wel akkoord<br>❑ Niet akkoord<br>❑ Helemaal niet akkoord
**1.E:** Euthanasie en palliatieve zorg excluderen elkaar.	❑ Helemaal akkoord<br>❑ Akkoord<br>❑ Niet/ wel akkoord<br>❑ Niet akkoord<br>❑ Helemaal niet akkoord
**1.F:** De psychiatrische patiënt is volgens mij wilsbekwaam.	❑ Helemaal akkoord<br>❑ Akkoord<br>❑ Niet/ wel akkoord<br>❑ Niet akkoord<br>❑ Helemaal niet akkoord
**1.G** Sinds de invoer van de euthanasiewetgeving denken patiënten vaak dat deze recht hebben op euthanasie (zowel bij fysisch en/ of psychisch ondraaglijk lijden). Volgens de patiënt is de arts dus moreel verplicht om in te stemmen met diens verzoek tot euthanasie. Gaat u hiermee akkoord?	❑ Helemaal akkoord<br>❑ Akkoord<br>❑ Niet/ wel akkoord<br>❑ Niet akkoord<br>❑ Helemaal niet akkoord

REEKS 2: voorkomen verzoek tot euthanasie op basis van psychisch ondraaglijk lijden:

**2.A:** Heeft een patiënt bij u ooit rechtstreeks een verzoek tot euthanasie, op basis van psychisch ondraaglijk lijden, geuit?	❑ Ja<br>❑ Nee (Ga naar vraag 2B)

Zo Ja: (**Opmerking:** indien frequentie >1: **u kan slechts 1 patiënt beschrijven.** kies hiervoor de meest recente.

**2.1** Frequentie	
Hoe vaak heeft dit al plaatsgevonden (voorkomen)?	❑ 1 keer<br>❑ 2 keer<br>❑ 3 keer<br>❑ > 3 keer
**2.2** Kenmerken van patiënt	
Wat was de leeftijd van die patiënt?	❑ <18<br>❑ 18-30<br>❑ 30-40<br>❑ 40-60<br>❑ >60
Geslacht van patiënt?	❑ Man<br>❑ Vrouw
Aandoening/ problematiek van de patiënt (hoofdklacht/ diagnose)?	❑ Schizofrenie (spectrum: schizo-affectief e.a.)<br>❑ Depressie (majeure)<br>❑ Depressie (dysthimie)<br>❑ Angststoornis<br>❑ Eetstoornis<br>❑ Persoonlijkheidsstoornis<br>❑ Bipolaire stoornis<br>❑ Verslavingsproblematiek<br>❑ Obsessief compulsieve stoornis<br>❑ Pervasieve ontwikkelingsstroonis<br>❑ ADHD (volwassenen)<br>❑ Andere (welke: _ _ _ _ _ _ _ _ _ _ _ _ _ _ _)
**2.3** Aanpak	
Heeft u het euthanasieverzoek met de patiënt verder besproken?	❑ Ja<br>❑ Nee
Hebt u dit verzoek genegeerd? (hierbij geldt ook dat het euthanasieverzoek beschouwd werd als suïcide wens).	❑ Ja<br>❑ Nee
Doorverwezen naar psychiater? (Niets zelf mee gedaan).	❑ Ja<br>❑ Nee
Heeft u dit besproken in team?	❑ Ja<br>❑ Nee
**2.4** Uitvoering	
Werd het euthanasieverzoek uitgevoerd (beoordeeld en na verloop van tijd uitgevoerd)?	❑ Ja<br>❑ Nee

**2.B:** Hebt u al gehoord van een mede-collega of een andere hulpverlener (bv psycholoog) dat een euthanasievraag vanuit de psychiatrische patiënt reeds is voorgekomen?	❑ Ja<br>❑ Nee
**2.C:** Hebt u al gehoord dat een euthanasieverzoek (binnen uw ziekenhuis) is uitgevoerd in praktijk?	❑ Ja<br>❑ Nee

REEKS 3: Attitude en visie verpleegkundige:

**3.A:** Bespreekbaar maken van euthanasie met de psychiatrische patiënt zou de doodswens kunnen versterken.	❑ Helemaal akkoord<br>❑ Akkoord<br>❑ Niet/ wel akkoord<br>❑ Niet akkoord<br>❑ Helemaal niet akkoord
**3.B:** Bespreekbaar maken van euthanasie met de psychiatrische patiënt zou de doodswens kunnen doen afnemen.	❑ Helemaal akkoord<br>❑ Akkoord<br>❑ Niet/ wel akkoord<br>❑ Niet akkoord<br>❑ Helemaal niet akkoord
**3.C:** Euthanasie op basis van ondraaglijk psychisch lijden is een vraag tot levenshulp.	❑ Helemaal akkoord<br>❑ Akkoord<br>❑ Niet/ wel akkoord<br>❑ Niet akkoord<br>❑ Helemaal niet akkoord
**3.D:** Euthanasie op basis van ondraaglijk psychisch lijden kan volgens mij beschouwd worden als aanwezige (passieve of actieve) suïcidegedachten. Er dient dus vooral suïcide preventie maatregelen genomen te worden.	❑ Helemaal akkoord<br>❑ Akkoord<br>❑ Niet/ wel akkoord<br>❑ Niet akkoord<br>❑ Helemaal niet akkoord

REEKS 4: Rol van de verpleegkundige (1):

**4.A:** Euthanasie bespreken is enkel een zaak tussen de psychiater en de patiënt.	❑ Helemaal akkoord<br>❑ Akkoord<br>❑ Niet/ wel akkoord<br>❑ Niet akkoord<br>❑ Helemaal niet akkoord
**4.B:** De psychiatrisch verpleegkundige kan en mag een euthanasievraag van de patiënt met hem/haar bespreken.	❑ Helemaal akkoord<br>❑ Akkoord<br>❑ Niet/ wel akkoord<br>❑ Niet akkoord<br>❑ Helemaal niet akkoord
**4.C:** Bij de beoordeling van het euthanasieverzoek is de input van de verpleegkundige die zorg draagt voor de patiënt van cruciaal belang.	❑ Helemaal akkoord<br>❑ Akkoord<br>❑ Niet/ wel akkoord<br>❑ Niet akkoord<br>❑ Helemaal niet akkoord

REEKS 5: Rol van de verpleegkundige (2):

**5.A:** Heeft u het gevoel om over voldoende informatie, kennis en/of vaardigheden te beschikken om met de euthanasievraag van de psychiatrische patiënt om te gaan?	❑ Ja<br>❑ Nee
**5.B:** Is huidig onderwerp in uw opleiding aan bod gekomen in het kader van geestelijke gezondheidszorg?	❑ Ja<br>❑ Nee<br>❑ Niet van toepassing want < 2002 was de euthanasiewetgeving nog niet goedgekeurd
**5.C:** Vindt u het belangrijk dat dit thema in de toekomst aan bod dient te komen in de verpleegkundige opleiding (binnen opleidingsonderdelen geestelijke gezondheidszorg)?	❑ Ja<br>❑ Nee

Mening vragenlijst:

Vond u deelname aan dit onderzoek nuttig?	❑ Ja<br>❑ Nee
Vond u dit onderzoek relevant voor dagelijkse praktijk?	❑ Ja<br>❑ Nee

Indien u dat wenst kan u hier opmerkingen noteren (vrijblijvend):

Bedankt voor uw tijd en deelname.
